# Gut microbiota variation between climatic zones and due to migration strategy in passerine birds

**DOI:** 10.3389/fmicb.2023.1080017

**Published:** 2023-02-01

**Authors:** Lucie Schmiedová, Jakub Kreisinger, Jan Kubovčiak, Martin Těšický, Jean-Francois Martin, Oldřich Tomášek, Tereza Kauzálová, Ondřej Sedláček, Tomáš Albrecht

**Affiliations:** ^1^Department of Zoology, Faculty of Science, Charles University, Prague, Czechia; ^2^Institute of Vertebrate Biology, Czech Academy of Sciences, Brno, Czechia; ^3^Montpellier SupAgro, Montferrier-sur-Lez, France; ^4^Department of Ecology, Faculty of Science, Charles University, Prague, Czechia

**Keywords:** faecal microbiome, gastrointestinal tract, metabarcoding, climatic zones, passerine birds

## Abstract

**Introduction:**

Decreasing biotic diversity with increasing latitude is an almost universal macroecological pattern documented for a broad range of taxa, however, there have been few studies focused on changes in gut microbiota (GM) across climatic zones.

**Methods:**

Using 16S rRNA amplicon profiling, we analyzed GM variation between temperate (Czechia) and tropical (Cameroon) populations of 99 passerine bird species and assessed GM similarity of temperate species migrating to tropical regions with that of residents/short-distance migrants and tropical residents. Our study also considered the possible influence of diet on GM.

**Results:**

We observed no consistent GM diversity differences between tropical and temperate species. In the tropics, GM composition varied substantially between dry and rainy seasons and only a few taxa exhibited consistent differential abundance between tropical and temperate zones, irrespective of migration behavior and seasonal GM changes. During the breeding season, trans-Saharan migrant GM diverged little from species not overwintering in the tropics and did not show higher similarity to tropical passerines than temperate residents/short-distance migrants. Interestingly, GM of two temperate-breeding trans-Saharan migrants sampled in the tropical zone matched that of tropical residents and converged with other temperate species during the breeding season. Diet had a slight effect on GM composition of tropical species, but no effect on GM of temperate hosts.

**Discussion:**

Consequently, our results demonstrate extensive passerine GM plasticity, the dominant role of environmental factors in its composition and limited effect of diet.

## Introduction

Microbial communities associated with animal hosts have a marked effect on the host’s physiology and immune system (Guarner and Malagelada, 2003; Sommer and Bäckhed, 2013). In the context of these modulatory effects, the gut microbiota (hereafter GM) inhabiting the lower intestine play a preeminent role. While GM cell counts are comparable with the number of cells in the host’s body (Sender et al., 2016), functional variation within the GM, in terms of gene count, is much higher (Qin et al., 2010). The GM has a crucial effect on the development of digestive tract morphology (Reikvam et al., 2011), is involved in the synthesis of essential bioactive molecules that cannot be synthesized by the host (Bäckhed et al., 2005), stimulates the host’s immune system (Macpherson and Harris, 2004; Wu and Wu, 2012), and protects against pathogens (Koch and Schmid-Hempel, 2011). Consequently, variation in GM composition contributes to differences in health status, body condition and other traits associated with host fitness (Moreno et al., 2003; Jumpertz et al., 2011; van Dongen et al., 2013).

Geographically segregated populations often show variations in GM (Gillingham et al., 2019; Grond et al., 2019); however, the factors driving this variation, as well as its consequences on the host, are not fully understood. Across the globe, both abiotic conditions and biotic interactions change with latitude, with potential effects on GM composition and diversity (Schemske et al., 2009). These include aerial temperature, humidity or solar radiation, which vary across climatic zones and can impact environmental bacterial sources of GM, but also the abundance and diversity of pathogens (Guernier et al., 2004; Nunn et al., 2005), which can modulate host GM through direct or indirect interactions with the host’s immune system (Lee et al., 2014; Kreisinger et al., 2015a). Last, but not least, variation in environmental conditions between climatic zones can impose indirect consequences on GM *via* selection of host phenotypic traits that affect colonization and proliferation of bacteria within the gut. In birds, high temporal stability and predictability of resources in tropical environments are associated with a comparatively higher life expectancy and a slower pace of life compared to phylogenetically related species inhabiting temperate climatic zones (Wiersma et al., 2007a,b). This has far-reaching effects on a plethora of ecological and life-history traits that show clear latitudinal trends across birds and may also affect the GM, along with reproductive investment, physiology and immunity (Stutchbury and Morton, 2001; Jetz et al., 2008; Andreani et al., 2020).

Unfortunately, there is a general scarcity of empirical studies on GM variation between climatic zones. To our knowledge, most of the relevant data available focused on human GM (De Filippo et al., 2010; Yatsunenko et al., 2012; Lee et al., 2014; Suzuki and Worobey, 2014, but see Suzuki et al., 2020), usually revealing marked variation in diversity and both taxonomic and functional GM composition between tropical and temperate human populations. However, these differences may have arisen as a consequence of contrasting human lifestyles in tropical and temperate zone environments, including differences in the proportion of energy-rich items in the diet (De Filippo et al., 2010), differences in sanitary conditions (Lee et al., 2014), or the use of antibiotics and other medicaments (Pérez-Cobas et al., 2013), rather than latitudinal contrast in environmental factors. Therefore, further research on non-human models is needed, in order to understand the contribution of environmental factors not directly linked with variation in lifestyle on GM structure across climatic zones.

To assess how GM varies between climatic zones, we applied a comparative approach based on 16S rRNA amplicon profiling of 99 bird species covering 37 passerine families, with species nesting both in temperate and tropical regions. Passerine birds represent the majority of avian species diversity and are a popular model group for research into ecological and life-history divergence between tropical and temperate organisms (Martin et al., 2000; Peach et al., 2001; Stutchbury and Morton, 2001; Wiersma et al., 2007b; Albrecht et al., 2013). There is an emerging interest in microbiota associated with avian hosts, including its interaction with avian ecology and physiology (Moreno et al., 2003; Kreisinger et al., 2015b,^2017^; Lewis et al., 2016b; Escallón et al., 2017). Though there have been a few studies analyzing avian GM in tropical populations (Hird et al., 2015; Bodawatta et al., 2018, 2021; Capunitan et al., 2020), none have directly compared tropical GM with temperate populations. In general, tropical areas are characterized by highly stable environmental conditions; nevertheless, periods of high and low precipitation (i.e., rainy vs. dry season) determine periodicity in many biological processes (e.g., reproductive season, migration, mounting or consumed diet). This contrast between the dry and rainy seasons could also impact GM (Gomez et al., 2016), though this possibility has never been addressed in birds. To fill this gap in knowledge, we included GM samples collected during both the dry and rainy seasons within our tropical samples.

Importantly, many temperate passerines migrate to tropical areas during the non-breeding season (Cepák et al., 2008), whereas others spend the whole year in temperate areas. Thus, the second aim of this study was to assess how migration behavior affects GM variation between passerine species. Environmental conditions at wintering grounds of long-distance migrants have a profound carry-over effect on a range of body condition traits expressed at breeding grounds (Norris et al., 2004; Saino et al., 2004; Rockwell et al., 2012). It is tempting to speculate that GM represents one of these carry-over effects and that exposure to environmental bacteria along migration routes and at wintering grounds could result in divergent GM structures between resident and migratory species during the breeding season. Alternatively, GM variation between migrating and resident species could be caused by ecological and physiological adaptations evolved as a consequence of migration behavior (Piersma et al., 1999; McWilliams and Karasov, 2001; Schwilch et al., 2002; Owen and Moore, 2008). Previous studies have already shown that avian GM can vary between breeding and wintering grounds (Wu et al., 2018), and that variation in migration behavior between closely related subspecies can affect GM (Turjeman et al., 2020). However, our study is the first to benefit from an extensive comparative dataset (comprising 52 species breeding in the temperate zone) that allows us to search for conserved GM patterns associated with long-distance migration that discriminate migrating and non-migrating species at their breeding grounds. Furthermore, a comparison of the GM profiles of two trans-Saharan migrant species at their wintering and breeding grounds, in the context of other syntopic passerine hosts, allowed us to assess GM turnover between temperate breeding grounds and wintering grounds in tropical areas.

## Materials and methods

### Field sampling

In this study, we analyzed faecal microbiota, which has been shown to be a good proxy for avian GM (Videvall et al., 2018; Berlow et al., 2020), with sample collection and storage as described in Kropáčková et al. (2017). Faecal samples of temperate passerines (405 samples from 52 species), were obtained during the 2014 breeding season (April–July) at various sampling sites in the Czechia (Supplementary Table 1 and Supplementary Figure 1) and were previously presented in Kropáčková et al. (2017). Tropical species (205 samples from 47 species) were sampled in upland forest habitats in Cameroon (Mount Cameroon; approximately 4°07′N, 9°04′E; Supplementary Table 1 and Supplementary Figure 1). Tropical samples were collected during both the rainy (September 2014 at 950 and 1 100 meters above mean sea level) and dry seasons (November/December 2014 at 650 and 2,280 meters above mean sea level), the latter corresponding to the breeding season for most tropical passerines included in our dataset. Furthermore, the migration and wintering period of temperate trans-Saharan migrants also largely overlaps with the dry season in Cameroon. During the dry season, we also collected 25 samples from two temperate-breeding trans-Saharan migrant species, the garden warbler (*Sylvia borin*) and willow warbler (*Phylloscopus trochilus*).

### Migration behavior, diet and host phylogeny

Temperate species were categorized as trans-Saharan migrants with wintering grounds in tropical sub-Saharan Africa (*n* = 148 samples and 19 species; Supplementary Figure 1) or residents/short-distance migrants (*n* = 257 samples and 33 species; Supplementary Figure 1) that do not fly as far as Sahara during their migration (Cepák et al., 2008). Most African species are sedentary or seasonal short-distance migrants and, while actual data on migration routes are mostly missing, it is unlikely that these species migrate over distances of more than 500 km (Fry et al., 1982). Data on diet for each species was extracted from the EltonTraits 1.0 database (Wilman et al., 2014) and classified on a continuous scale ranging from full herbivory/granivory to full insectivory/carnivory. We considered diet as a predictor of gut microbiota because it is recognized as the main factor for microbial community formation in mammals, whereas its role in birds is still controversial (Capunitan et al., 2020; Bodawatta et al., 2021). The effect of diet may vary also between climatic zones, as dietary specialization is expected to be higher in tropical hosts (Stutchbury and Morton, 2001). No other ecological variables were included in the statistical models as they are often unknown for tropical species. In addition, our previous research suggests a very low effect of host ecology on GM variation compared to phylogenetic relatedness and geography (Kropáčková et al., 2017).

To account for phylogenetic co-variance, a set of 1,000 Bayesian trees with Hackett backbone was prepared for the species sampled (Jetz et al., 2012).^1^ Subsequently, a maximum clade credibility tree was constructed using the maxCladeCred function in the R package phangorn (Schliep, 2011).

### Microbiota profiling

Metagenomic DNA from faecal samples was extracted using the PowerSoil DNA isolation kit (MO BIO Laboratories Inc., Carlsbad, CA, USA). Primers covering the V3-V4 variable region on bacterial 16S rRNA [i.e., S-D-Bact-0341-b-S-17 (CCTACGGGNGGCWGCAG) and S-D-Bact-0785-a-A-21 (GACTACHVGGGTATCTAATCC); both tagged by 10 bp barcodes] were used during the PCR step (Klindworth et al., 2013). For the polymerase chain reaction (PCR), we used 8 μl of KAPA HIFI Hot Start Ready Mix (Kapa Biosystems, Wilmington, MA, USA), 0.37 μM of each primer and 7 μl of DNA template. PCR conditions were as follows: initial denaturation at 95°C for 5 min followed by 35 cycles, each of 98°C (20 s), 61°C (15 s) and 72°C (40 s), and a final extension at 72°C for 5 min. The PCR products were subsequently pooled at equimolar concentration and purified using the High Pure PCR Product Purification Kit (Roche, Switzerland). Sequencing adaptors were ligated using TruSeq nano DNA library preparation kits (Illumina, San Diego, CA, USA) and the resulting amplicon libraries were sequenced on a single Miseq run (Illumina, San Diego, CA, USA) using v3 chemistry and 2 × 300 bp paired-end reads. We also sequenced 34 blank isolates along with the GM samples and used these for the identification of putative bacterial contaminates. A detailed description of the laboratory procedures is provided in Kropáčková et al. (2017). All laboratory procedures were completed within 1 year after sample collection.

### Bioinformatic processing of 16S rRNA data

Sample demultiplexing and detection and trimming of gene-specific primers were undertaken using Skewer (Jiang et al., 2014). Reads of low quality, i.e., those with an expected error rate per paired-end read >2, were then eliminated. Dada2 (Callahan et al., 2016) was used for denoising of quality-filtered reads and subsequent quantification of 16S rRNA amplicon sequence variants (hereafter ASVs) in each sample. Chimeric ASVs were detected and eliminated using UCHIME (Edgar et al., 2011) and gold.fna, a chimera-free reference database.^2^ Using the Decontam package (Davis et al., 2018), we identified and subsequently eliminated 69 putatively contaminating ASVs whose prevalence was increased in blank isolates compared to GM samples and/or showed greater representation in samples with a low concentration of metagenomic DNA (as assessed based on concentration of PCR products). Furthermore, we excluded ASVs assigned as “Chloroplast” (18.1% of reads after quality filtering), “Mitochondria” (7.2% of reads after quality filtering), or those not assigned to any bacterial phylum (<0.01% of reads after quality filtering) from all downstream analyses. Subsequently, we clustered all remaining ASVs at the 97% similarity threshold using vsearch (Rognes et al., 2016) and assigned taxonomy of representative sequences for each of the 3,281 resulting operational taxonomic units (OTUs) using RDP classifier (>0.5 posterior confidence; Wang et al., 2007) and the Silva reference database (v.138; Quast et al., 2013). Representative OTU sequences were further aligned using DECIPHER (Wright, 2020), the maximum likelihood tree being constructed using FastTree (Price et al., 2009). The final dataset comprised 6.98 million high-quality sequences (median number of reads per sample = 8,353, range = 1,006–138,465).

### Statistical analysis

Gut microbiota alpha diversity was assessed using Shannon indices, the number of observed OTUs and phylogenetic diversity (Faith, 1992). All the diversity indices were calculated for individual samples after OTU table rarefaction (i.e., random sub-setting of read counts per sample corresponding to minimal sequencing depth; 1 006 sequences/sample). To assess variation in GM composition, we calculated both Bray-Curtis and a binary version of Jaccard dissimilarity between samples. Jaccard dissimilarity only accounts for OTU presence/absence and, therefore, is more sensitive than Bray-Curtis dissimilarity to GM changes driven by rare OTUs. The resulting dissimilarity matrices were used as an input for principal coordinate analysis (PCoA), the PCoA ordination being used for visual inspection of variation in GM composition. Scores for the first two PCoA axes were later used as response variables in statistical models of variation in GM composition. Our data had a complex structure where non-independence of samples due to repeated sampling of the same species had to be taken into account and where, at the same time, the composition of the gut microbiota could also reflect the phylogeny of the hosts. This prevented us from using PERMANOVA or similar tools widely used in microbial ecology that include a dissimilarity matrix as a response, because of the limited ability of these models to flexibly account for different sources of non-independence in the data.

We applied bootstrap-based tests to assess differences in GM composition between the four categories of passerine host (i.e., tropical from rainy or dry season and temperate trans-Saharan migrants or residents/short-distance migrants). Specifically, for each pair of host categories (e.g., dry vs. rainy season tropical hosts), we extracted two vectors of GM dissimilarity corresponding either to different host species from the same category or to different host species from different categories. Dissimilarities for same host species pairs were not considered. Next, we calculated the mean difference between these two vectors and corresponding 95% bootstrap-based confidence intervals using the two.boot() function in the R package simpleboot (*n* = 1,000 resampling steps; Peng, 2019). In parallel, we analyzed the effect of climatic zone, migration and diet on GM alpha diversity and composition using generalized linear mixed models (GLMM), with phylogenetic correlations fitted using the R package phyloglmm (Li and Bolker, 2019). In the case of GM alpha diversity, diversity indices were used as the GLMM response, with observed OTU counts log_10_ transformed to achieve normal distribution of residuals. In the case of GM compositional variation, we considered either PCoA scores for GM dissimilarity matrices or abundances (i.e., read counts) of individual OTUs in each sample as response variables. The models estimated correlation due to host phylogeny, while systematic variation among species was modeled *via* random effects. All models, except those focused on OTU abundance, were assumed to have a Gaussian distribution. Models based on OTU abundance were assumed to have a negative binomial distribution, while the log-scaled total number of sequences per sample was considered as an offset to account for uneven sequencing depth. To achieve convergence between the OTU-specific models, each model was only fitted for OTUs detected in >20 samples and represented by >500 sequences in total. Significance testing associated with GLMMs was based on type II sum of squares, meaning that the effect of climatic zone, migration and season was adjusted for the effect of diet and *vice versa*. In the case of OTU-level analyses, false discovery rates (FDR; Benjamini and Hochberg, 1995) were calculated to account for multiple testing and only effects with an FDR <0.05 were reported as significant. Furthermore, we applied Tukey *post-hoc* tests for comparisons between the four host species categories, i.e., tropical samples from dry and rainy seasons, temperate zone trans-Saharan migrants and residents/short-distance migrants. The effect of host phylogeny was assessed based on a comparison of GLMMs with and without phylogenetic correlation. Similarly, the effect of host species identity was assessed based on a comparison of models including host species as a random effect vs. a simplified model ignoring information on host species. Tropical samples from the dry and rainy season were collected at different altitudes, which could confound the effect of seasonality. At the same time, we were unable to include these two variables in a single statistical model due to poor convergence of corresponding GLMMs. Therefore, to assess the importance of altitude and seasonality on alpha and beta diversity of GM variation in tropical passerines, we fitted GLMMs for a subset of tropical samples that included the effect of season or altitude as an explanatory variable and controlled for the effect of diet. We then compared these GLMM pairs using the Akaike Information Criterion (AIC). All statistical analyses were undertaken using the R statistical environment v.4.0.3 (R Core Team, 2020).

## Results

### Alpha diversity variation

**FIGURE 1:**
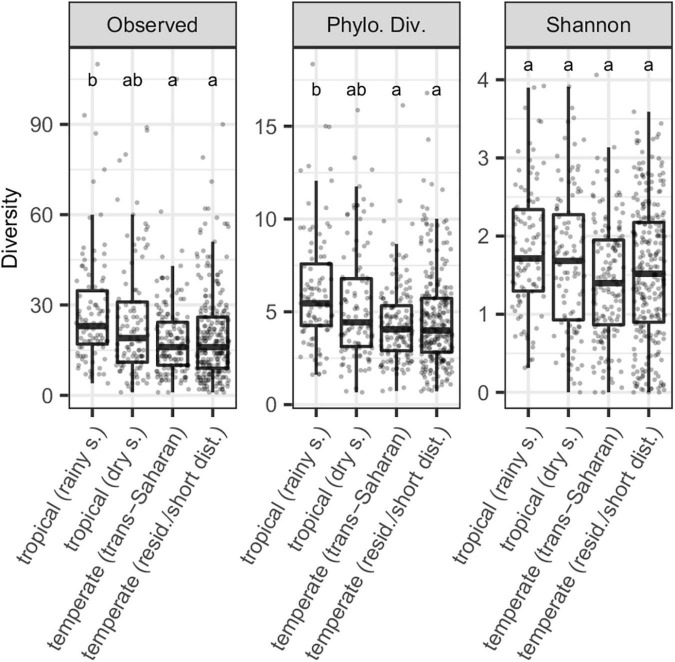
Gut microbiota (GM) alpha diversity variation for three diversity measures (number of observed OTUs, phylogenetic diversity and Shannon diversity) in tropical passerines sampled during the wet or rainy season, temperate trans-Saharan migrants and temperate residents/short-distance migrants. Different letters above the bars indicate significant differences between the two host groups according to Tukey *post-hoc* tests (*p* < 0.05), whereas the presence of at least one common letter indicates a non-significant difference.

While the number of OTUs and phylogenetic diversity were highest in tropical birds during the rainy season, this was not true for Shannon diversity. At the same time, there was no significant difference in alpha diversity between dry season tropical birds and trans-Saharan migrants or residents/short-distance migrants sampled in the temperate zone (Figure 1, Table 1, and Supplementary Table 2). Furthermore, all GM alpha diversity measurements, except for the Shannon index, increased with the increasing proportion of insects in the diet (Supplementary Figure 2). GLMMs on alpha diversity accounting for host phylogeny and species identity received higher support than more simplified versions lacking phylogenetic correlations and/or species identity random effects, suggesting that host species identity, as well as their phylogenetic relatedness, had an impact on GM diversity (Supplementary Table 3).

**TABLE 1:** Parameter estimates for mixed models analyzing the effect of climatic zones, season, migration, and diet on GM alpha diversity.

Response	Predictor	Estimate	Standard error	Z statistic	*P-*value
Shannon	(Intercept)	1.5683	0.2763	5.6760	0.0000
	Tropical (Dry S.)	−0.0740	0.1273	−0.5814	0.5609
	Temperate (Trans Saharan)	−0.3434	0.1425	−2.4089	0.0160
	Temperate (Resid, Short)	−0.2890	0.1429	−2.0217	0.0432
	Diet	0.3273	0.2013	1.6259	0.1040
Observed	(Intercept)	4.2685	0.5167	8.2611	0.0000
	Tropical (Dry S.)	−0.2183	0.2444	−0.8933	0.3717
	Temperate (Trans Saharan)	−0.8621	0.2857	−3.0181	0.0025
	Temperate (Resid, Short)	−0.7914	0.2884	−2.7440	0.0061
	Diet	0.9590	0.4001	2.3968	0.0165
Phylog. div	(Intercept)	2.1131	0.1850	11.4198	0.0000
	Tropical (Dry S.)	−0.1130	0.0875	−1.2908	0.1968
	Temperate (Trans Saharan)	−0.3605	0.1016	−3.5497	0.0004
	Temperate (Resid, Short)	−0.2979	0.1014	−2.9390	0.0033
	Diet	0.3564	0.1419	2.5114	0.0120

Three alpha diversity measures (Shannon diversity, number of observed OTUs and phylogenetic diversity) were used as a response.

Parameter estimates, standard error, z statistics and corresponding *p*-values associated with individual models are shown in the table. All models included species identity as a random factors and estimated phylogenetic correlation among species.

Neither altitude nor season had a significant effect on GM alpha diversity in a subset of the tropical samples. Consequently, models with altitude as a predictor did not receive higher support than models on seasonal variation (Supplementary Table 4).

### Whole community GM divergence

**FIGURE 2:**
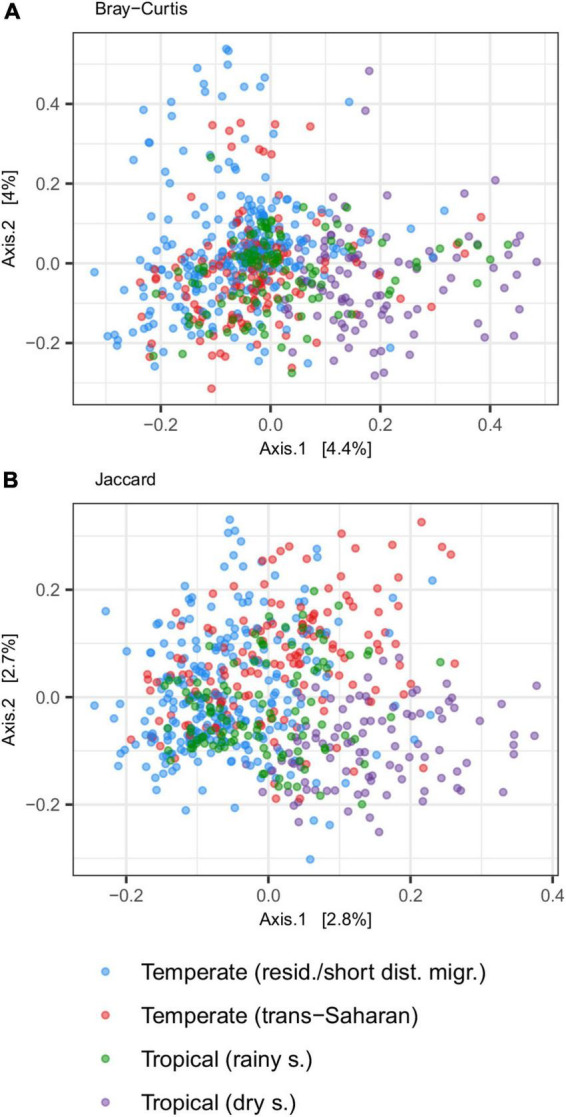
Gut microbiota (GM) differentiation among tropical passerines collected during dry or rainy season and trans-Saharan migrants or residents/short-distance migrants collected in temperate zone according to PCoA ordination. PCoA was performed on two types of community distances [**(A)** Jaccard and **(B)** Bray-Curtis].

**FIGURE 3:**
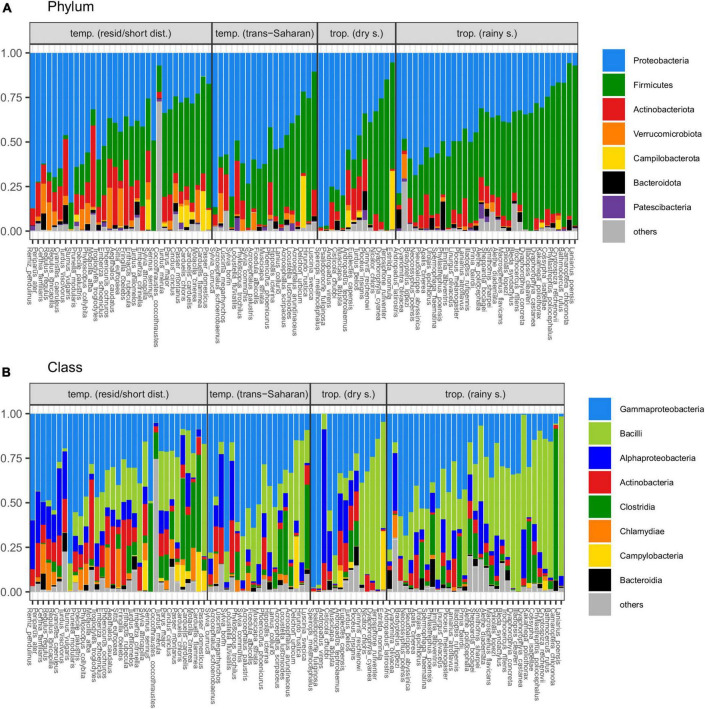
Average proportions of dominating bacterial **(A)** phyla and **(B)** classes in gut microbiota (GM) of individual passerine species (“others” indicates taxa representing <1% of whole community).

Principal coordinate analysis revealed considerable overlap in GM composition between the four passerine host groups (Figure 2). Nevertheless, consistent with patterns in alpha diversity, subsequent GLMMs for the first two ordination axes suggested that tropical passerines from rainy season harbor the most distinct GM composition compared to other host groups (Table 2, Supplementary Table 5, and Supplementary Figure 3). According to taxonomic barplots (Figure 3), this difference was largely driven by an increase in the abundance of the phylum Firmicutes during the rainy season, which represented, on average, 49.5% of reads in tropical hosts during the rainy season, 32.4% during the dry season, 34.9% in temperate trans-Saharan migrants, and 32.3% in temperate residents/short-distance migrants (Supplementary Table 6). Aside from tropical samples during the rainy season, GLMMs revealed significant variation between the other three host groups, with the magnitude and significance of these differences being dependent on the combination of the actual GM dissimilarity index and the PCoA axis. In addition, GLMMs also uncovered a significant link between GM and diet composition (Supplementary Figure 4). The effect of phylogeny on GM composition was supported in the case of GLMMs for the first PCoA axis of Bray-Curtis dissimilarity and the second PCoA axis of Jaccard dissimilarity. Models completely ignoring both the effects of interspecific variation and phylogeny exhibited much lower performance than more complex GLMMs (Supplementary Table 7).

**TABLE 2:** Parameter estimates for mixed models testing the effect of climatic zones, season, migration, and diet on GM composition.

Response	Predictor	Estimate	Standard error	Z statistic	*P*-value
Bray-Curtis axis 1	(Intercept)	−0.0097	0.0604	−0.1598	0.8731
	Tropical (Rainy S.)	0.1319	0.0164	8.0362	0.0000
	Temperate (Resid, Short)	−0.0784	0.0267	−2.9383	0.0033
	Temperate (Trans Saharan)	−0.0779	0.0268	−2.9108	0.0036
	Diet	0.0896	0.0402	2.2270	0.0259
Jaccard axis 1	(Intercept)	−0.0421	0.0172	−2.4526	0.0142
	Tropical (Rainy S.)	0.1342	0.0141	9.5085	0.0000
	Temperate (Resid, Short)	−0.0496	0.0139	−3.5629	0.0004
	Temperate (Trans Saharan)	0.0003	0.0156	0.0210	0.9833
	Diet	0.0666	0.0199	3.3468	0.0008
Bray-Curtis axis 2	(Intercept)	0.0105	0.0244	0.4320	0.6657
	Tropical (Rainy S.)	0.0066	0.0204	0.3241	0.7458
	Temperate (Resid, Short)	0.0534	0.0197	2.7112	0.0067
	Temperate (Trans Saharan)	0.0201	0.0220	0.9146	0.3604
	Diet	−0.0664	0.0282	−2.3564	0.0185
Jaccard axis 2	(Intercept)	−0.0255	0.0420	−0.6075	0.5435
	Tropical (Rainy S.)	−0.0697	0.0156	−4.4699	0.0000
	Temperate (Resid, Short)	0.0270	0.0201	1.3385	0.1807
	Temperate (Trans Saharan)	0.0839	0.0201	4.1812	0.0000
	Diet	0.0017	0.0307	0.0541	0.9569

Scores for the first and the second PCoA axis calculated based on two dissimilarity indexes (Bray-Curtis and Jaccard) were used as response variables.

Parameter estimates, standard error, z statistics, corresponding *p*-values associated with individual models are shown.

Separate PCoAs and subsequent GLMMs for the temperate species subset revealed significant variation between trans-Saharan migrants and residents/short-distance migrants based on Jaccard, but not Bray-Curtis, dissimilarity, though the effect of diet was not significant (Supplementary Table 8). On the other hand, both GM dissimilarity indices uncovered a significant effect of diet on the tropical passerine subset (Supplementary Table 8). The effect of season was found to be a better predictor of GM variation for the subset of tropical passerines than the effect of altitude (Supplementary Table 4). In addition to PCoA, bootstrap analysis was applied to elucidate the effect of climatic zone and migration on GM composition. This approach used raw dissimilarity values between host species and not PCoA ordination of all GM samples and, as such, provides more straightforward insights into effect sizes of GM differences between host categories. On the other hand, this routine does not account directly for the effect of diet and phylogeny, and does not utilize information on intraspecific GM variation. By using the bootstrap approach, we found that the highest differences in GM composition were between both temperate species categories and tropical species from the rainy season. Seasonal GM variation in the tropical zone tended to be higher than the difference between temperate and tropical GM collected during the dry season. Temperate zone residents/short-distance migrants did not exhibit higher dissimilarity to tropical birds than trans-Saharan migrants. Indeed, the opposite was true in the case of Bray-Curtis dissimilarity. These analyses also suggested low difference between trans-Saharan migrants and temperate zone residents/short-distance migrants, with corresponding confidence intervals overlapping with zero (Supplementary Figure 5).

### Bacterial OTUs involved in GM divergence

Differential abundance analysis for the 121 dominant OTUs (74% of all reads) identified 40 OTUs (41.5% of all reads) whose abundances were affected by climatic zone, migration or a variation between dry vs. rainy seasons. More specifically, 19 OTUs in tropical hosts varied between the dry and rainy seasons and the abundance of three OTUs varied between trans-Saharan migrants and temperate zone residents/short-distance migrants. In addition, there was pronounced variation between temperate and tropical hosts sampled both during the dry (13 and 16 OTUs varied in trans-Saharan migrants and residents/short-distance migrants, respectively) and rainy seasons (significant difference for 25 and 23 OTUs, respectively; Supplementary Figure 6). Finally, two OTUs from the genus *Rickettsia* and family Rhizobiales were positively correlated with the proportion of insects in the diet. OTU-level GLMM parameter estimates of contrasts between dry season tropical birds and trans-Saharan migrants were strongly correlated with contrasts between dry season tropical samples and temperate residents/short-distance migrants (Pearson correlation: *r* = 0.9610, *p* < 0.0001), with no significant difference in their values (Paired *t*-test: *t* = −0.8022, *p* = 0.4273). The same held true for the comparison of rainy season tropical samples with the two groups of temperate hosts (Pearson correlation: *r* = 0.8684, *p* < 0.0001; Paired *t*-test: *t* = 0.8022, *p* = 0.4273). This suggests, that tropical environment does not directly affect GM of trans-Saharan migrants during their breeding season.

As PCoA-based GLMMs revealed a pronounced effect of diet in a subset of tropical passerines, but no effect of diet in the temperate zone, we conducted additional differential abundance analyses on corresponding subsets, the results of which showed that no OTUs were associated with diet in temperate zone passerines but five OTUs that were more abundant in insectivorous passerines in the tropical zone (Supplementary Figure 7).

### GM of trans-Saharan migrants at wintering and breeding grounds

**FIGURE 4:**
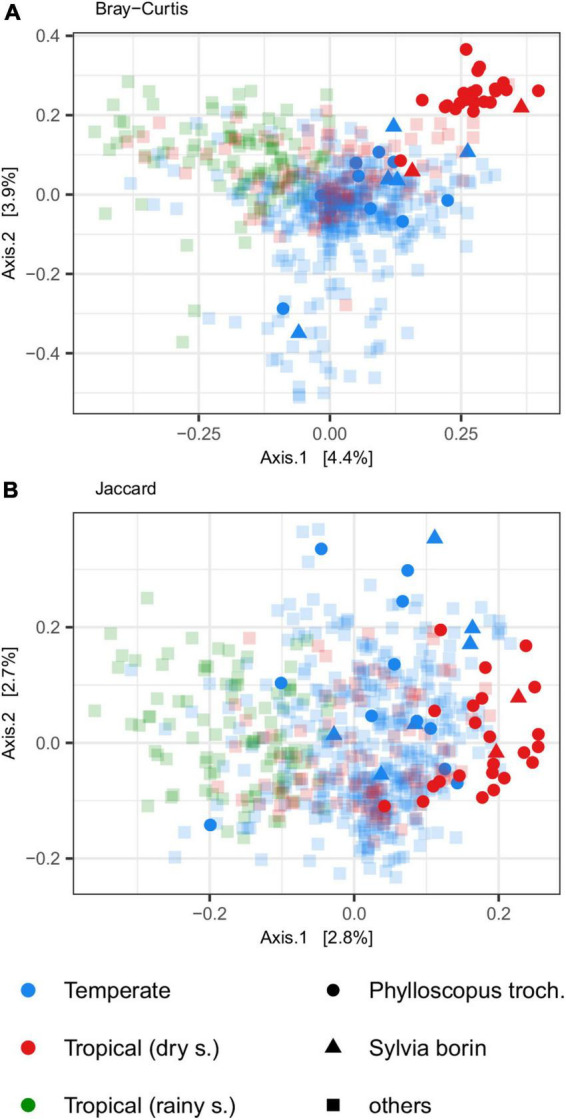
Principal coordinate analysis (PCoA) for **(A)** Bray-Curtis and **(B)** Jaccard dissimilarities depicting gut microbiota (GM) changes between two trans-Saharan species (willow warbler–*Phylloscopus trochilus* and garden warbler–*Sylvia borin*; in opaque colors) that were collected in temperate zone during the breeding period (blue) or tropical zone during the migration and wintering period (red). Samples from other species are indicated by semitransparent squares.

**FIGURE 5:**
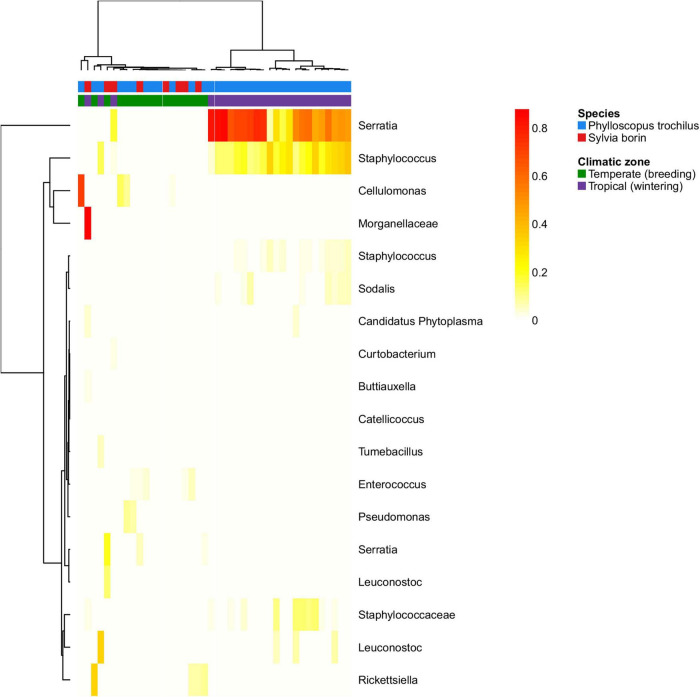
Heatmap for relative abundances of OTUs that exhibited significant changes between breeding vs. wintering grounds of two temperate trans-Saharan migrants (willow warbler–*Phylloscopus trochilus* and garden warbler–*Sylvia borin*). Matrix was clustered using Ward method.

To assess GM changes between breeding and wintering grounds in trans-Saharan migrants, we sampled the GM of the garden warblers (*S. borin*) and the willow warblers (*P. trochilus*) in the temperate zone during their breeding season (*n* = 11 and 6, respectively) and in the tropical region during their migration and wintering period (*n* = 23 and 2, respectively). PCoA suggested a dramatic effect of tropical and temperate environments on GM composition in both species. PCoA ordination showed that, while GM samples collected at their breeding grounds showed perfect overlap with other temperate zone passerines, their GM changed dramatically in the tropics. The direction of this change was congruent with the overall direction of the difference between temperate species and tropical passerines from the dry season (Figure 4). At the same time, tropical and temperate environments had no effect on GM diversity of the two species (GLM with Gaussian distribution: *p* > 0.5 in both cases; Supplementary Figure 8). The negative binomial models implemented in the DESeq2 package (Love et al., 2014), which account for variation between host species, showed that the relative abundances of 16 OTUs varied between the breeding and wintering grounds (Figure 5), with the most prominent changes detected in the case of *Serratia* and *Staphylococcus* OTUs.

## Discussion

### GM of tropical passerines during the rainy and dry seasons

While the tropical environment is generally considered to be relatively stable and predictable, annual fluctuations in precipitation between the dry and rainy seasons can affect a plethora of biological processes, including reproduction, migration and feather mounting (Cox et al., 2013; Nwaogu et al., 2019). These periodic changes can also impact GM, as has been shown for great apes, where GM composition undergoes remarkable changes due to seasonal shifts in the diet (Gomez et al., 2016). Though several studies have been undertaken on avian GM in tropical regions (Hird et al., 2015; Bodawatta et al., 2018, 2021; Capunitan et al., 2020), the variation between dry and rainy seasons has not yet been examined in any detail. Our comparative dataset uncovered substantial changes in passerine GM composition and alpha diversity between rainy and dry seasons within the same geographic location, with the changes being of a comparable, or even more pronounced effect size as GM variation between temperate and tropical host populations separated by >5,000 km. This suggests that actual environmental conditions at a given locality have a decisive effect on passerine GM. At the proximate level, the observed changes could be linked with a range of different mechanisms, such as environmental sources of GM bacteria being affected by seasonal variation in abiotic conditions. The diet consumed is also likely to vary between the dry and rainy seasons, and thus could contribute to the observed difference in GM. Last but not least, physiological and behavioral aspects associated with reproduction could also play some role in seasonal GM changes as reproduction of most tropical passerines in our study area mainly takes place during the dry season (Serle, 1981; Tye, 1992; Fotso, 1996). Unfortunately, we still know very little about how these factors contribute to GM composition in birds. Likewise, the role of seasonal variation of these factors in the tropical environment is not sufficiently understood. As such, further research is required to uncover the mechanistic basis of temporal fluctuations in avian GM in tropical environments.

### GM variation between climatic zones

A decrease in biotic diversity with increasing latitude is a universal macroecological pattern that has been observed across a broad range of taxa (McCoy and Connor, 1980; but see Owen and Owen, 1974), including parasites and pathogens associated with animal hosts (Guernier et al., 2004). To date, however, the diversity of host-associated microbial communities has rarely been investigated in this context, with the most relevant data being based on GM profiling in human populations (De Filippo et al., 2010; Yatsunenko et al., 2012; Lee et al., 2014; Suzuki and Worobey, 2014; but see Suzuki et al., 2020). Most of these studies provided support for an increase in GM diversity in tropical compared with temperate populations (De Filippo et al., 2010; Lee et al., 2014; but see Suzuki and Worobey, 2014). However, these differences were probably caused by contrasting lifestyles and environments between developed and developing countries. This is also supported by a recent observation that the GM of people from developed countries that stay for a long period in tropical areas and adopt a local lifestyle, converge rapidly with those of local residents (Gomez et al., 2016).

The results of our comparative study do not suggest any pronounced systematic effect of climatic zone on passerine GM alpha diversity as tropical species exhibited significantly increased GM diversity compared to temperate zone passerines during the rainy season only. Despite the lack of any clear difference in GM alpha diversity, community dissimilarity analysis revealed substantial shifts in GM composition between temperate and tropical passerines, with GM profiles from the rainy season exhibiting greater dissimilarity to temperate passerines than GM collected during the dry season. However, subsequent differential abundance analysis revealed that the divergence between temperate and tropical hosts collected during dry and rainy seasons was predominantly determined by different sets of OTUs. At the same time, only four OTUs exhibited consistent differential abundance between tropical species, regardless if sampled during dry or rainy season, and temperate species, irrespective of their migration behavior. The OTUs exhibiting consistently higher abundance in temperate hosts comprised *Rickettsiella* and *Rickettsia* that represent insect pathogens (Jurat-Fuentes and Jackson, 2012) and, insect-transmitted obligate intracellular parasites of vertebrate respectively (Abdad et al., 2018). The OTU from the genus *Methylobacterium-Methylorubrum*, generally assumed to be of environmental origin (Green and Ardley, 2018), was also more abundant in temperate passerines. A single OTU from the genus *Bradyrhizobium*, comprising predominantly soil-dwelling species that are often involved in mutualistic interactions with plants (Ormeño-Orrillo and Martínez-Romero, 2019), showed a significant decrease in abundance in temperate species (compared with tropical hosts.), irrespective of the season in which samples were collected or host migration behavior. As all OTUs having consistent variation between tropical and temperate species exhibited a tight association with insects or were likely of environmental origin, we assume that GM differences between climatic zones were mainly affected by a divergence in the bacterial pool present in the diet and other environmental resources. Conversely, our results show that the contribution of contrasting ecology and life history-linked phenotypic traits in tropical vs. temperate passerines to GM variation between climatic zones appears to be of relatively low importance. This does not mean that host ecology does not affect GM, however, nor that its contribution to GM variation cannot vary between climatic zones. Indeed, as differential abundance analysis detected five OTUs positively linked with the proportion of insects in diet of tropical passerines, and no OTUs affected by diet in temperate hosts, our data suggest that diet has a greater effect on the GM of tropical passerines. Nevertheless, these results should be interpreted with caution. Alternative explanations for the observed patterns may lie in the fact that the dietary data we used do not reflect flexibility in foraging habits that can be greater in temperate species. Indeed, temperate zone passerines frequently change their diet opportunistically at different times of the year and according to resource availability (Carter et al., 2021). Furthermore, the diet of temperate long-distance migrants is more dependent on insects, which further complicates statistical separation of the effect of diet and migration.

### Effect of migration

In this study, we adopted two approaches to untangle how migration behavior affects GM in passerines. First, we studied GM changes between the breeding and wintering grounds of two trans-Saharan migrant species (garden warbler and willow warbler) to assess the actual effect of climatic zone on GM. Second, we conducted a comparative analysis using GM sampled during the breeding season for temperate passerines that varied in migration behavior, allowing us to evaluate long-lasting pervasive effects of migration behavior on GM.

The GM of the two species of trans-Saharan migrants (collected at breeding grounds in the temperate zone and at dry season in the tropics during their wintering period) exhibited substantial differences in composition, but not in alpha diversity. According to PCoA sample ordination, these changes corresponded with overall GM differentiation between temperate zone passerines and tropical passerines sampled during the dry season, but not the rainy season. Moreover, a *Staphylococcus* OTU, which contributed most to GM compositional change between wintering and breeding grounds, was also more abundant in tropical species during the dry season than during the rainy season. Altogether, our data suggest that environment is the main driver of GM variation between wintering and breeding grounds in trans-Saharan migrant species. Consistent with our results, several previous studies have reported variation in GM composition between breeding and wintering grounds, or during spring and autumn migrations in other migratory birds (Lewis et al., 2016a,b; Wu et al., 2018; Zhang et al., 2020). However, none of these previous studies took the opportunity to contrast corresponding GM profiles with an extensive comparative dataset that included other species residing at the breeding and wintering grounds. Consequently, our study is the first to allow an assessment of whether observed changes between breeding and wintering grounds reflect GM variation in other co-occurring bird species at corresponding sites.

In addition to spatially diversified pools of environmental microbes interacting with the GM of long-distance migrants, behavioral, physiological and ecological adaptations associated with migration (Piersma et al., 1999; McWilliams and Karasov, 2001; Schwilch et al., 2002; Owen and Moore, 2008) could also have specific impacts on migrant GM. It is tempting to speculate that all these factors could have a long-lasting pervasive effect on the GM composition of long-distance migrants, making it distinct from that of resident species cohabiting the same geographic area.

According to our comparative analysis comprising 19 species of trans-Saharan migrants and 33 temperate residents/short-distance migrants, however, the GM of trans-Saharan migrants during the breeding season showed only a small, though still significant, difference to that of other temperate species. Furthermore, our data suggest that these differences are unlikely to be caused by bacteria incorporated into GM during overwintering and migration as alpha diversity of trans-Saharan migrants was not increased compared to other temperate zone passerines and GM composition did not show a lower dissimilarity than tropical hosts. Moreover, only one (genus *Lactococcus*) of three OTUs over-represented in the subset of all trans-Saharan migrants at their breeding grounds (compared to other temperate zone species) exhibited higher abundance in tropical hosts during the dry season than in temperate residents/short-distance migrants. Finally, two OTUs, exhibiting a striking increase in abundance in garden and willow warblers at their wintering grounds, did not vary between trans-Saharan migrants and other temperate passerines at their breeding grounds. Altogether, the results indicate that GM acquired at wintering grounds and during migration rapidly converge to a GM typical for the temperate zone following their spring arrival at the breeding grounds. This is in line with our previous study demonstrating a low level of GM stability over time in another trans-Saharan migrant, the barn swallow (*Hirundo rustica*; Kreisinger et al., 2017). Similarly, Risely et al. (2017, 2018) showed that difference in GM between young non-migrating individuals and adult migrants of a non-passerine bird, the red-necked stint (*Calidris ruficollis*), tended to decline gradually after returning from their wintering grounds.

Compared to temperate residents/short-distance migrants, the GM of trans-Saharan migrants at breeding grounds was characterized by an increased abundance of three OTUs of lactic acid bacteria (LAB, genus *Carnobacterium*, *Enterococcus*, and *Lactococcus*) that prefer energy-rich substrates and are capable of fermenting carbohydrates under anoxic conditions (Kandler, 1983). The presence of some LAB is believed to be generally beneficial as they stimulate the host’s immune system and produce metabolites involved in the maintenance of GM homeostasis (Ljungh and Wadström, 2006). Moreover, some LAB species contribute to the host’s energy balance *via* improved feed utilization (Abe et al., 1995), which can be particularly beneficial for long-distance migrants adapted for energy-demanding migration, often associated with a considerable shortage of food. As the diet of migratory passerines typically comprises a higher percentage of insects, we intentionally adjusted our comparative analysis to include this confounding variable. Nevertheless, owing to the above-mentioned issues associated with dietary data, we cannot fully guarantee that our analysis precisely separated the effects of migration and diet. It is also worth noting that the OTU from the genus *Lactococcus* over-represented in our trans-Saharan migrants was also more abundant in insectivores from the tropical zone.

## Conclusion

Our study provides the first insight into GM variation between tropical and temperate passerines. We show that GM composition and diversity differ dramatically between the dry and rainy seasons in tropical hosts and, consequently, only a limited number of bacterial OTUs exhibit consistent differential abundance between tropical and temperate zones, irrespective of seasonal GM changes in the tropics. These OTUs mostly correspond to insect-borne or environmental bacteria, suggesting a predominant impact of environment on GM differentiation across climatic zones. In trans-Saharan migrants, we observed a dramatic difference in GM between wintering and breeding grounds, consistent with overall GM differentiation of other passerines inhabiting these two regions. At the same time, systematic differences between trans-Saharan migrants and temperate residents or short-distance migrants at breeding grounds were relatively low and were probably not caused by bacteria incorporated into GM at wintering grounds or during migration. Diet had a relatively low effect and our data suggest that it has a greater effect on the GM of tropical hosts compared to temperate hosts. Altogether, our results demonstrate extensive passerine GM plasticity and the dominating role of environmental factors on its composition.

## Data availability statement

Sequencing data are available at the European Nucleotide Archive under project accession number: PRJEB53462. The scripts associated with this study and a database of processed 16S rRNA data and sample metadata can be found at https://github.com/jakubkreisinger/GM_trop_temp.

## Ethics statement

This animal study was reviewed and approved by all field procedures were conducted in accordance with the Guidelines for Animal Care and Treatment of the European Union, and approved by the Animal Care and Use Committees at the Czech Academy of Sciences (041/2011), and Charles University in Prague (4789/2008-0).

## Author contributions

JKr and TA: study design. TA, MT, OT, TK, and OS: field sampling. LS and JKu: laboratory analysis. JKr and LS: data analysis and manuscript drafting. TA, JKr, and JKu: funding. All authors provided helpful comments and recommendations and approved the final version of the manuscript.

## Footnotes

1
http://birdtree.org/

2
https://drive5.com/uchime/gold.fa
