# Supplementary material for: Gut microbiota variation between climatic zones and due to migration strategy in passerine birds
Source: Front Microbiol. 2023 Feb 1;14:1080017. doi: 10.3389/fmicb.2023.1080017 (PMC9928719; doi:10.3389/fmicb.2023.1080017)

Supplementary Material

Gut microbiota variation between climatic zones and due to migration strategy in passerine birds

Schmiedová L[1], Kreisinger J[1,*], Kubovčiak J[1], Těšický M[1], Martin JF[3], Tomášek O[1,2], Kauzálová T[2], Sedláček O[4], Albrecht T[1,2]

*** Correspondence:** Corresponding Author: jakubkreisinger@seznam.cz

1 Supplementary Figures

Supplementary Figure 1: Phylogenetic tree of species included in the study. Geographic distribution and migration behaviour is indicated by different colours.

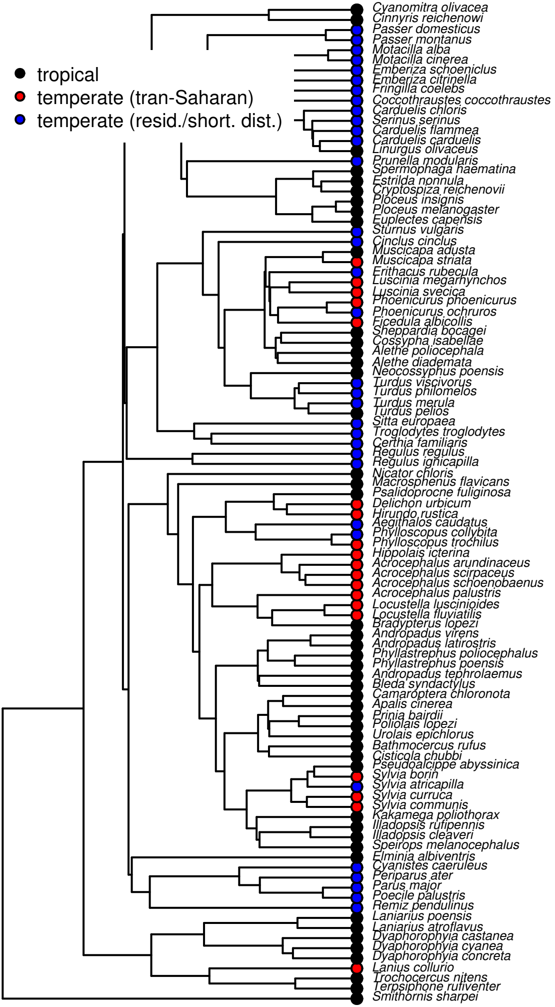

tropical
temperate (tran-Saharan)
temperate (resid./short. dist.)
Cyanomitra olivacea
Cinnyris reichenowi
Passer domesticus
Passer montanus
Motacilla alba
Motacilla cinerea
Emberiza schoeniclus
Emberiza citrinella
Fringilla coelebs
Coccothraustes coccothraustes
Carduelis chloris
Serinus serinus
Carduelis flammea
Carduelis carduelis
Linurgus olivaceus
Prunella modularis
Spermophaga haematina
Estrilda nonnula
Cryptospiza reichenovii
Ploceus insignis
Ploceus melanogaster
Euplectes capensis
Sturnus vulgaris
Cinclus cinclus
Muscicapa adusta
Muscicapa striata
Erithacus rubecula
Luscinia megarhynchos
Luscinia svecica
Phoenicurus phoenicurus
Phoenicurus ochruros
Ficedula albicollis
Sheppardia bocagei
Cossypha isabellae
Alethe poliocephala
Alethe diademata
Neocossyphus poensis
Turdus viscivorus
Turdus philomelos
Turdus merula
Turdus pelios
Sitta europaea
Troglodytes troglodytes
Certhia familiaris
Regulus regulus
Regulus ignicapilla
Nicator chloris
Macrosphenus flavicans
Psalidoprocne fuliginosa
Delichon urbicum
Hirundo rustica
Aegithalos caudatus
Phylloscopus collybita
Phylloscopus trochilus
Hippolais icterina
Acrocephalus arundinaceus
Acrocephalus scirpaceus
Acrocephalus schoenobaenus
Acrocephalus palustris
Locustella luscinioides
Locustella fluviatilis
Bradypterus lopezi
Andropadus virens
Andropadus latirostris
Phyllastrephus poliocephalus
Phyllastrephus poensis
Andropadus tephrolaemus
Bleda syndactylus
Camaroptera chloronota
Apalis cinerea
Prinia bairdii
Poliolais lopezi
Urolais epichlorus
Bathmocercus rufus
Cisticola chubbi
Pseudoalcippe abyssinica
Sylvia borin
Sylvia atricapilla
Sylvia curruca
Sylvia communis
Kakamega poliothorax
Illadopsis rufipennis
Illadopsis cleaveri
Speirops melanocephalus
Elminia albiventris
Cyanistes caeruleus
Periparus ater
Parus major
Poecile palustris
Remiz pendulinus
Laniarius poensis
Laniarius atroflavus
Dyaphorophyia castanea
Dyaphorophyia cyanea
Dyaphorophyia concreta
Lanius collurio
Trochocercus nitens
Terpsiphone rufiventer
Smithornis sharpei

Supplementary Figure 2: Association of GM alpha diversity with proportion of insects in diet. Three alpha diversity measures were analysed, namely number of observed OTUs, phylogenetic diversity and Shannon diversity. Probability values based in Type II sum of squares associated with each alpha diversity measure are shown. Parameter estimates are presented in Supplementary Table 2.

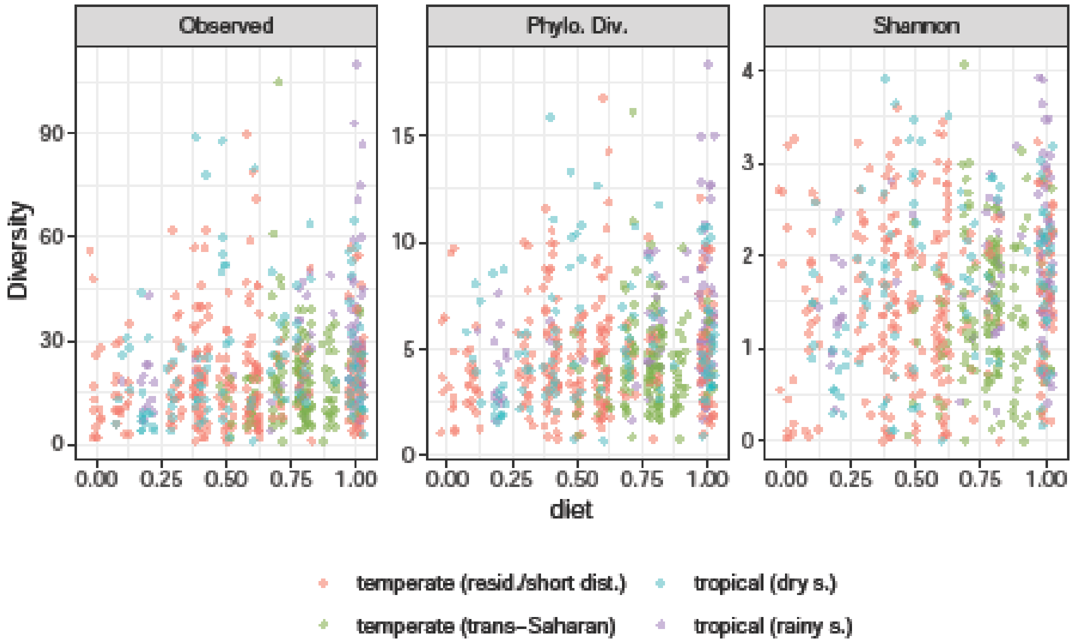

Supplementary Figure 3: Variation in PCoA scores for first two ordination axes between tropical passerines collected during dry or rainy season and trans-Saharan migrants or residents/short-distance migrants collected in temperate zone. Different letters above the bars indicate significant differences between the two host groups according to Tukey post hoc tests ($p < 0.05$), whereas the presence of at least one common letter indicates a nonsignificant difference.

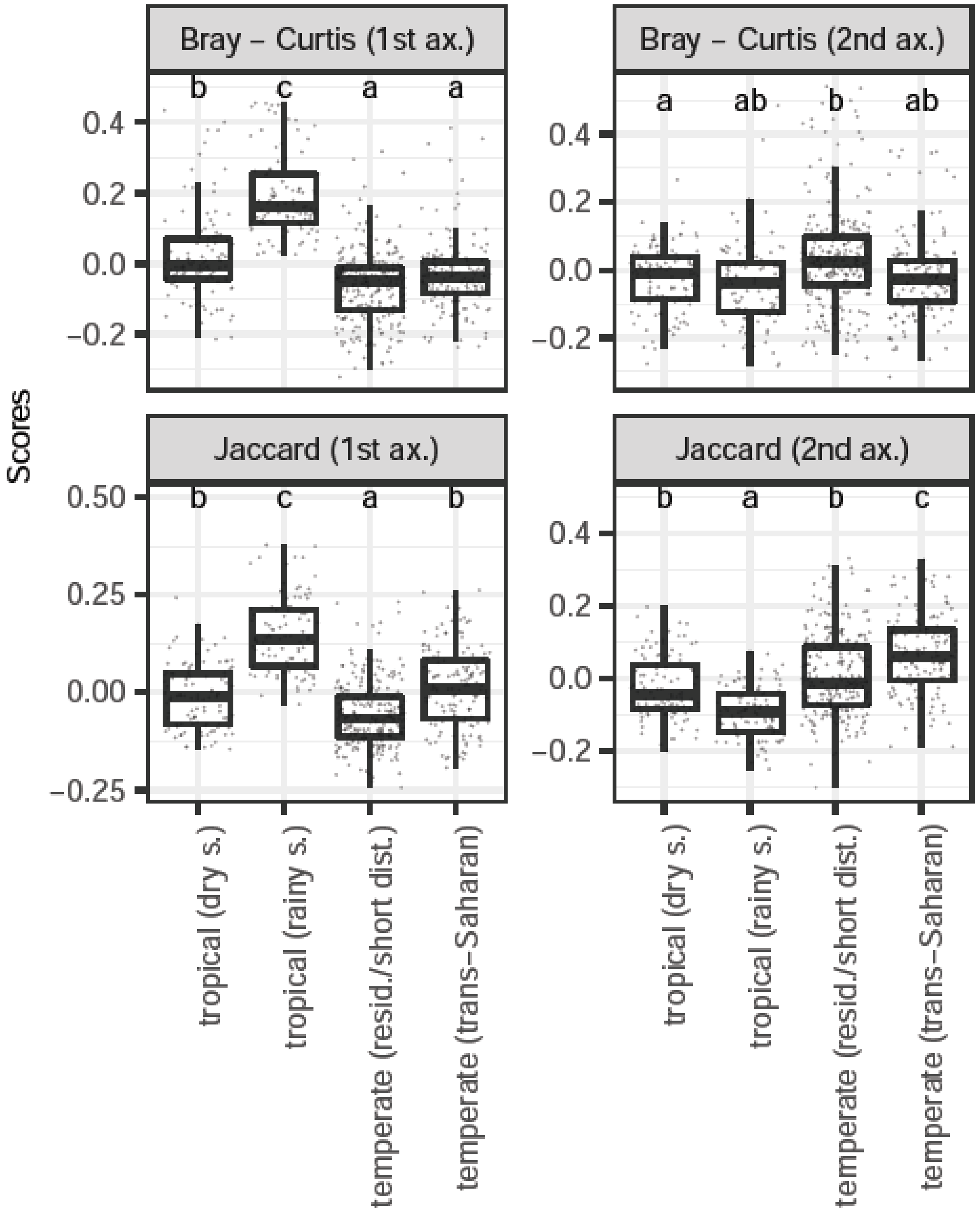

Supplementary Figure 4: Association of GM composition (i.e. scores for the first two PCoA axes) with proportion of insects in diet. PCoA was conducted for Bray-Curtis and Jaccard dissimilarities. Probability values based in Type II sum of squares associated with response measure are shown. Parameter estimates are presented in Supplementary Table 5.

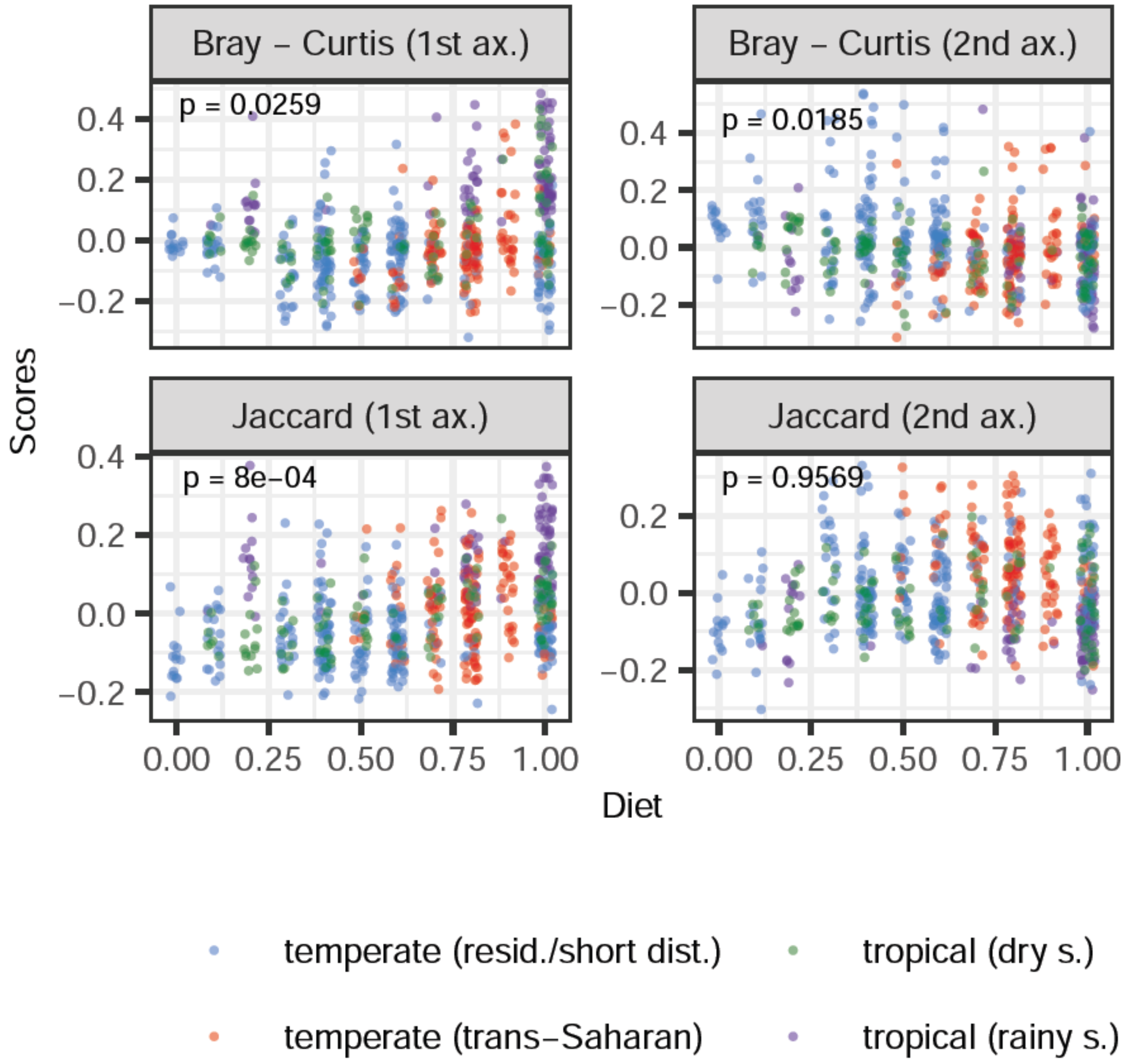

Supplementary Figure 5: Average difference in interspecific dissimilarities for species belonging to the same vs. the opposite category. Error bars correspond to 95% bootstrap-based confidence intervals**.**

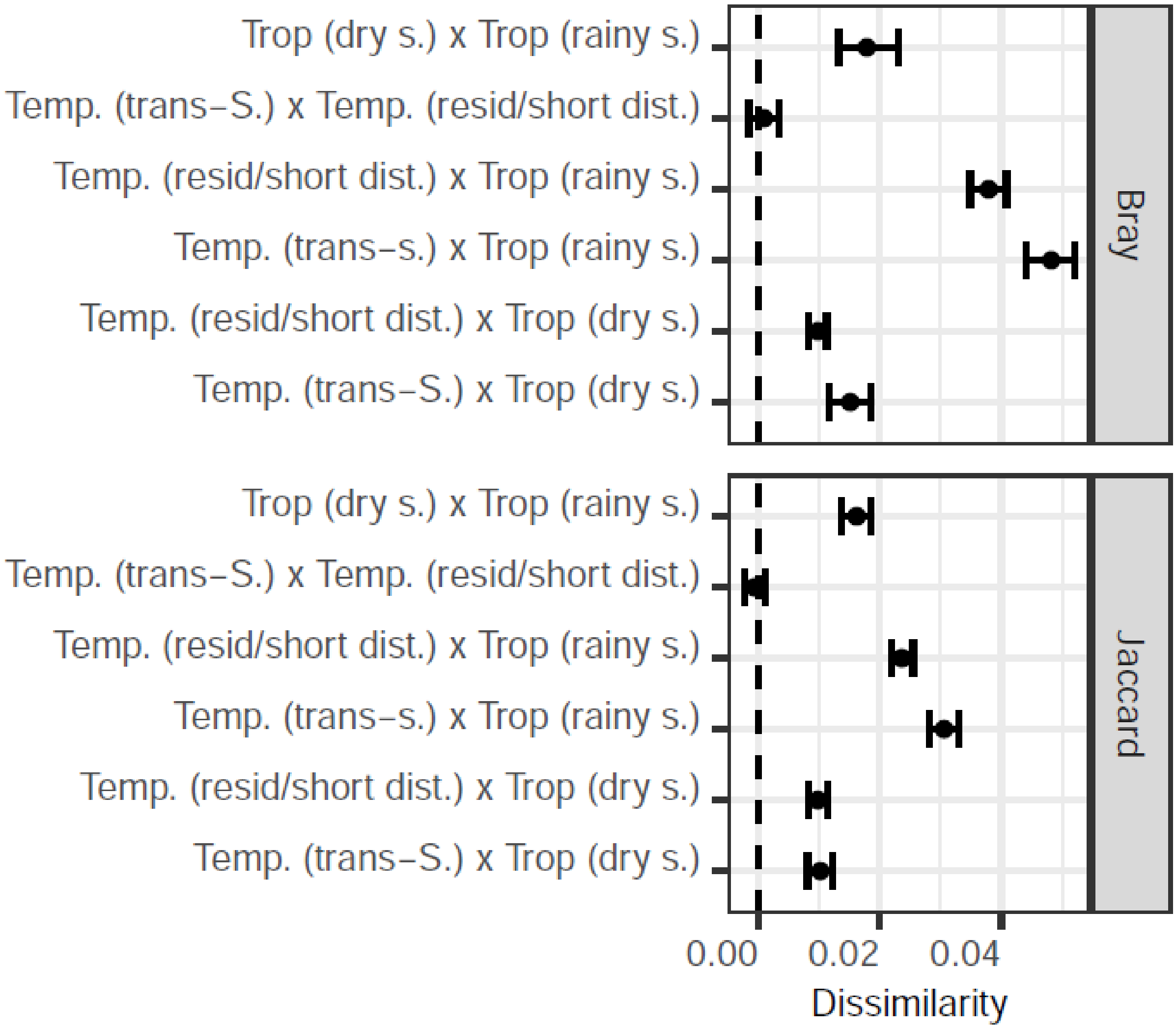

Supplementary Figure 6: OTUs that exhibited significant variation between four categories of passerine hosts according to GLMMs for data with negative binomial distribution. Only OTUs that passed multiple testing corrections (FDR < 0.05) are shown. Different letters above the bars indicate significant differences between the two host groups according to Tukey post hoc tests ($p < 0.05$), whereas the presence of at least one common letter indicates a nonsignificant difference.

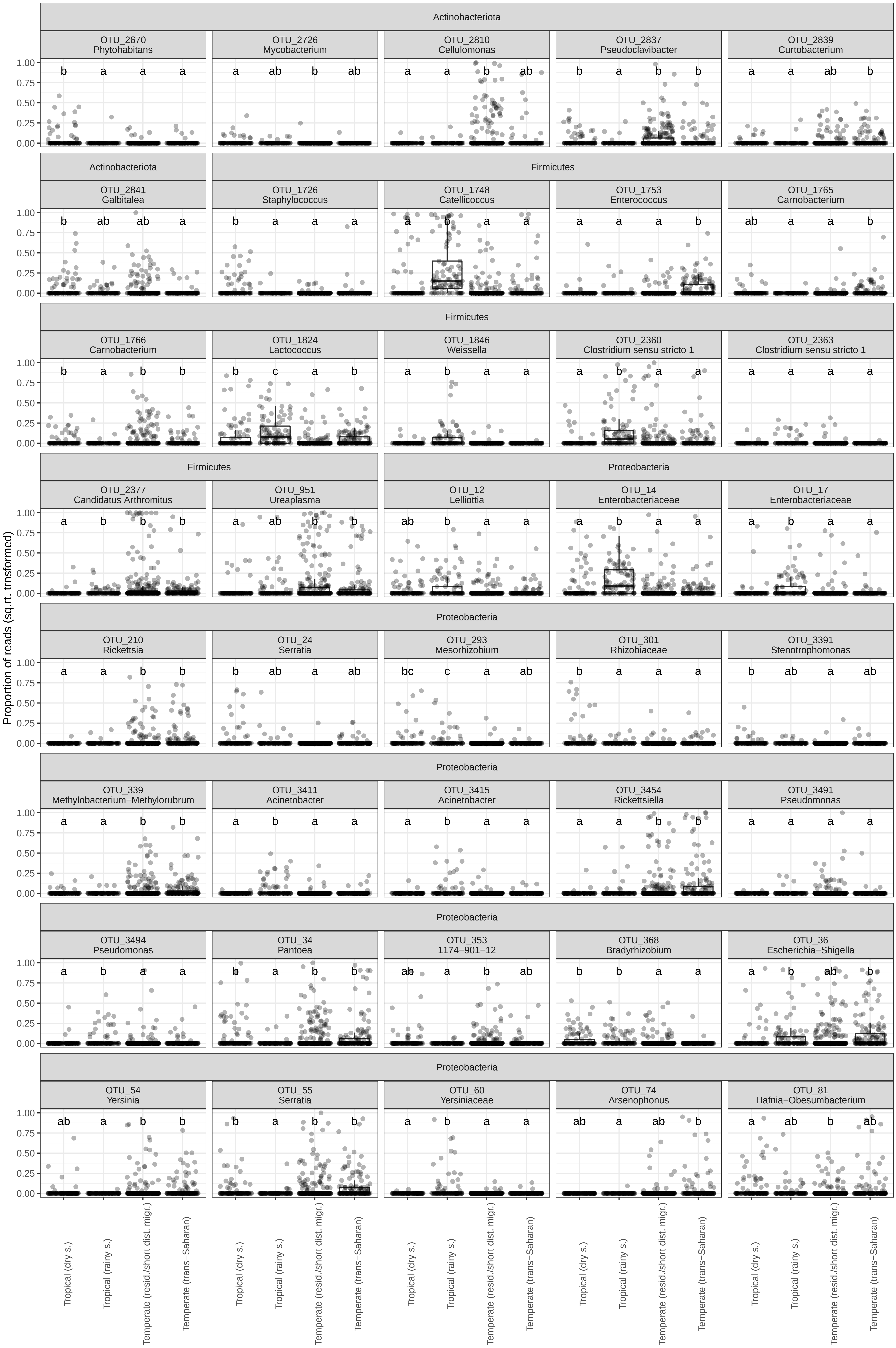
Actinobacteriota
OTU_2670 Phytohabitans
b a a a
OTU_2726 Mycobacterium
a ab b ab
OTU_2810 Cellulomonas
a a b ab
OTU_2837 Pseudoclavibacter
b a b b
OTU_2839 Curtobacterium
a a ab b
Actinobacteriota
OTU_2841 Galbitalea
b ab ab a
Firmicutes
OTU_1726 Staphylococcus
b a a a
OTU_1748 Catellicoccus
a b a a
OTU_1753 Enterococcus
a a a b
OTU_1765 Carnobacterium
ab a a b
Firmicutes
OTU_1766 Carnobacterium
b a b b
OTU_1824 Lactococcus
b c a b
OTU_1846 Weissella
a b a a
OTU_2360 Clostridium sensu stricto 1
a b a a
OTU_2363 Clostridium sensu stricto 1
a a a a
Firmicutes
OTU_2377 Candidatus Arthromitus
a b b b
OTU_951 Ureaplasma
a ab b b
Proteobacteria
OTU_12 Lelliottia
ab b a a
OTU_14 Enterobacteriaceae
a b a a
OTU_17 Enterobacteriaceae
a b a a
Proteobacteria
OTU_210 Rickettsia
a a b b
OTU_24 Serratia
b ab a ab
OTU_293 Mesorhizobium
bc c a ab
OTU_301 Rhizobiaceae
b a a a
OTU_3391 Stenotrophomonas
b ab a ab
Proteobacteria
OTU_339 Methylobacterium−Methylorubrum
a a b b
OTU_3411 Acinetobacter
a b a a
OTU_3415 Acinetobacter
a b a a
OTU_3454 Rickettsiella
a a b b
OTU_3491 Pseudomonas
a a a a
Proteobacteria
OTU_3494 Pseudomonas
a b a a
OTU_34 Pantoea
b a b b
OTU_353 1174−901−12
ab a b ab
OTU_368 Bradyrhizobium
b b a a
OTU_36 Escherichia−Shigella
a b ab b
Proteobacteria
OTU_54 Yersinia
ab a b b
OTU_55 Serratia
b a b b
OTU_60 Yersiniaceae
a b a a
OTU_74 Arsenophonus
ab a ab b
OTU_81 Hafnia−Obesumbacterium
a ab b ab
Proportion of reads (sq.rt. trnsformed)
1.00
0.75
0.50
0.25
0.00
Tropical (dry s.)
Tropical (rainy s.)
Temperate (resid./short dist. migr.)
Temperate (trans−Saharan)

Supplementary Figure 7: OTUs that exhibited significant association with diet on a subset of tropical samples according to GLMMs data with negative binomial distribution. Only OTUs that passed multiple testing corrections (FDR < 0.05) are shown.

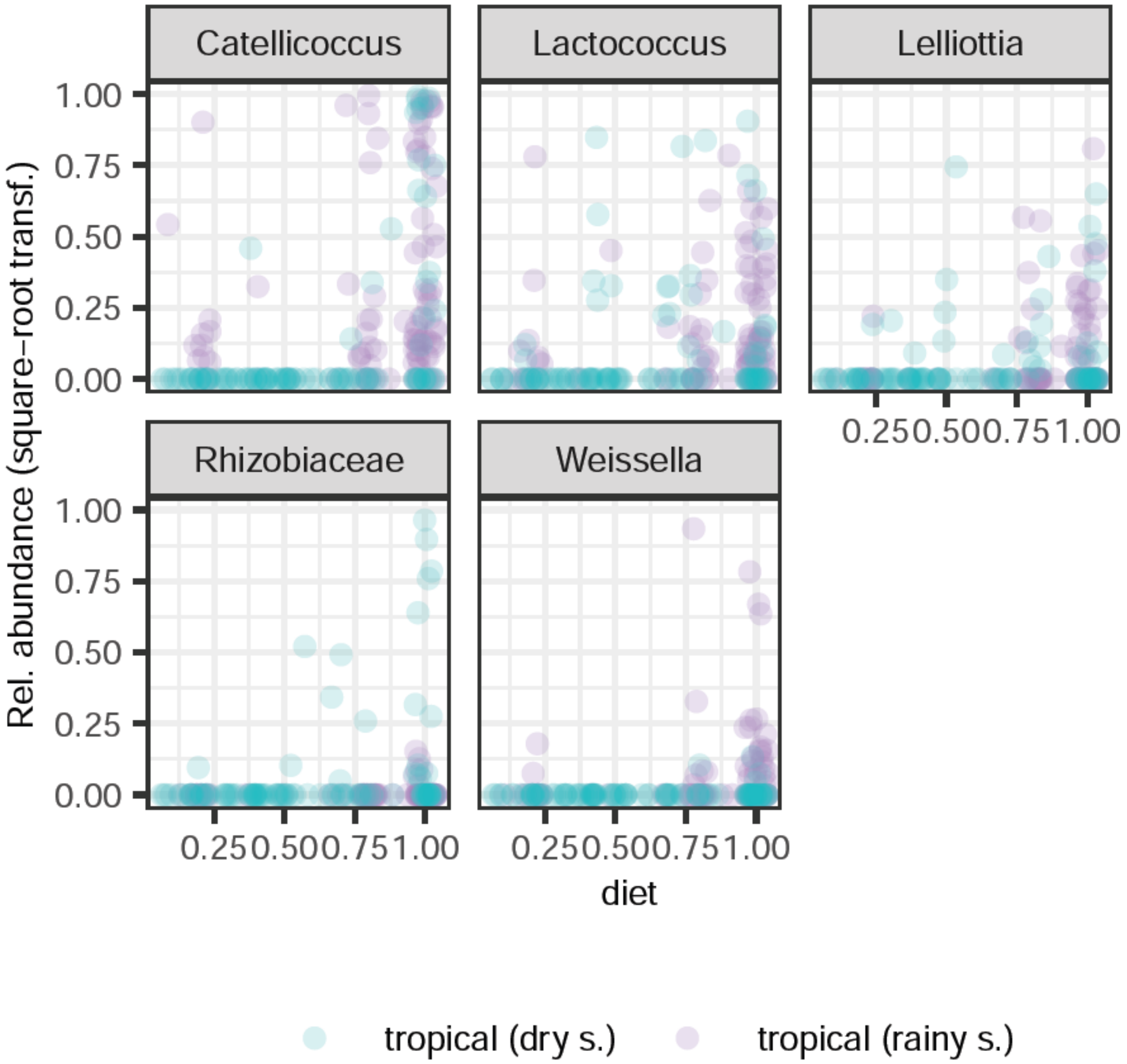

Supplementary Figure 8: Alpha diversity variation between breeding vs. wintering grounds of two temperate trans-Saharan migrants (willow warbler - *Phylloscopus trochilus* and garden warbler - *Sylvia borin*)**.**

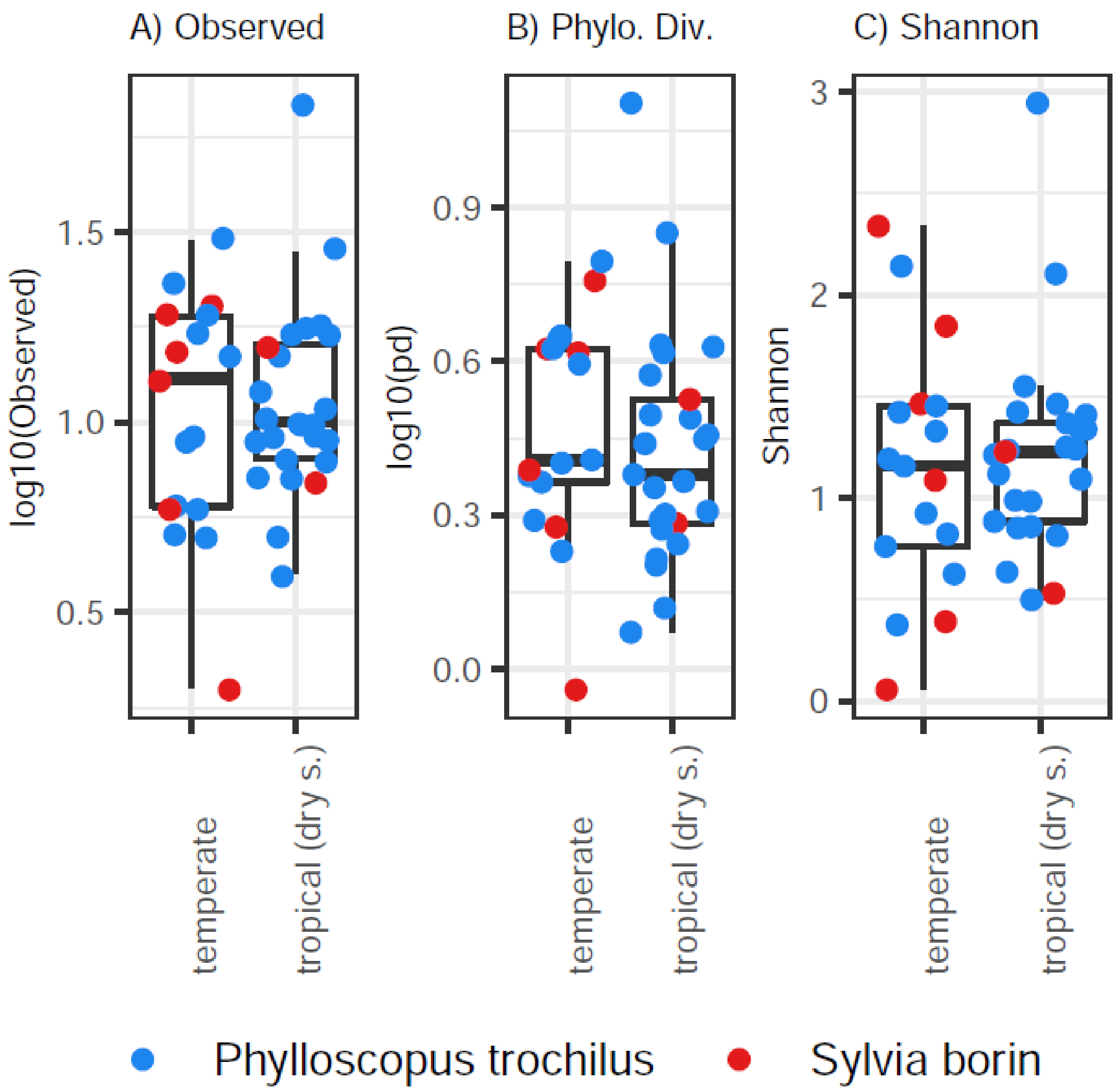

Supplement: Supplementary file 1 [file Data_Sheet_1.pdf]
